# Preparation and Application of Directed Vat Set Indigenous Freeze-Drying *Lentilactobacillus hilgardii* Q19 Starter in Winemaking

**DOI:** 10.3390/foods12051053

**Published:** 2023-03-01

**Authors:** Ling Wang, Gang Huang, Wen Ma, Gang Jin

**Affiliations:** 1 School of Agriculture, Ningxia University, Yinchuan 750021, China; 2 School of Food and Wine, Ningxia University, Yinchuan 750021, China; 3 Engineering Research Center of Ministry of Grape and Wine, Yinchuan 750021, China

**Keywords:** *Lentilactobacillus hilgardii*, vacuum freeze-drying, lyoprotectants, response surface methodology, volatile compounds

## Abstract

In order to prepare a better direct vat set for malolactic fermentation (MLF) in high ethanol and low pH wines, the high-ethanol- and low-temperature-tolerant strain *Lentilactobacillus hilgardii* Q19, which was isolated from the eastern foothill of the Helan Mountain wine region in China, was used to prepare a direct vat set by vacuum freeze-drying. A superior freeze-dried lyoprotectant was obtained to create the starting culture by selecting, combining, and optimizing numerous lyoprotectants with higher protection for Q19 by using a single-factor experiment and response surface approach. Finally, the *Lentilactobacillus hilgardii* Q19 direct vat set was inoculated in Cabernet Sauvignon wine to carry out MLF on a pilot scale, with commercial starter culture Oeno1 as control. The volatile compounds, biogenic amines, and ethyl carbamate content were analyzed. The results showed that a combination of 8.5 g/100 mL skimmed milk powder, 14.5 g/100 mL yeast extract powder, and 6.0 g/100 mL sodium hydrogen glutamate offered better protection; with this lyoprotectant, there were (4.36 ± 0.34) × 10^11^ CFU/g cells after freeze-drying, and it showed an excellent ability to degrade L-malic acid and could successfully finish MLF. In addition, in terms of aroma and wine safety, compared with Oeno1, the quantity and complexity of volatile compounds were increased after MLF, and biogenic amines and ethyl carbamate were produced less during MLF. We conclude that the *Lentilactobacillus hilgardii* Q19 direct vat set could be applied as a new MLF starter culture in high-ethanol wines.

## 1. Introduction

Malolactic fermentation (MLF) is the process by which lactic acid bacteria (LAB) decarboxylate L-malate into L-lactic acid and release CO_2_. It usually occurs after the completion of alcohol fermentation (AF). This process reduces the acidity of the wine and improves the complexity of volatile compounds, the wine’s microbial stability, and quality [1,2]. Spontaneously initiated MLF, which depends on the bacteria present in the wine environment, is usually challenging to control and cannot even start MLF in some cases [3,4]. Commercial LAB is primarily used for MLF in wines, also known as a directed vat set (DVS). DVS refers to a highly concentrated and standardized starter culture that has been prepared by vacuum-packing after the target strain has been cultured at high density, cultured, isolated, mixed well with protective agents, and vacuum freeze-dried. [5]. It is widely used in MLF because of its advantages, such as convenient inoculation, high viable count, easily controlled fermentation process, and long shelf life. Recently, several emerging drying technologies have been developed for the production of lactic acid bacteria DVS starters, such as low-temperature spray-drying technology [6], freezing granulation drying technology [7], and spray freeze-drying [8], which help to address the problem of low survival rates.

The eastern foothill of Helan Mountain in China has a vast wine-grape planting area with abundant microbial resources. The development and use of indigenous malolactic bacteria (MLB) provides a natural tolerance in indigenous wine fermentation environments, while simultaneously maintaining the original flavor substances in wine [9]. In this way, wine-producing areas can retain their characteristics and reduce production costs. On the other hand, in the wine-producing areas in the west of China, most of the MLF is conducted in November and December with low temperatures. For some wineries with poor temperature-control conditions, there will be some difficulties in using traditional LAB for MLF. *L. hilgardii* Q19, used in this experiment, has the characteristics of high-alcohol resistance and low-temperature fermentation [10]. It is a unique solution to this problem. Therefore, the development of *L. hilgardii* Q19 starter culture is highly significant. Currently, there are many methods for producing LAB as starters, for example, vacuum-drying, spray-drying, vacuum freeze-drying, and freezing bacteria in liquid nitrogen, but vacuum freeze-drying is a more convenient and widespread method to prepare LAB as starters [11,12]. It can maintain the thallus’ physiological, biochemical characteristics, and biological activity and adequately preserve the strains. However, this method exposes LAB to adverse conditions, such as drying, low temperature, and a vacuum environment, which may easily cause LAB mechanical damage, cell membrane damage, and DNA damage [13,14,15]. Therefore, the survival rate of LAB after freeze-drying is reduced. Meanwhile, the reasonable selection and use of lyoprotectants can effectively increase the freeze-dried survival rate [16].

In this study, based on high-density cultures, the effects of various lyoprotectants (skimmed milk powder, yeast extract powder, D-trehalose dihydrate, D-sorbitol, glycerol, sucrose, and L-Glutamic acid sodium salt) and their ratios on the survival rate of *L. hilgardii* Q19 during vacuum freeze-drying were investigated by single-factor experiments. A response surface optimization test was carried out on a pilot scale to deduce the most effective formulation of the freeze-dried protective agent and prepare a higher viable number of *L. hilgardii* Q19 starter. The volatile components and biogenic amine content of wine after MLF were analyzed to prepare a favorable MLF starter culture, increase the diversity of DVS starters, and provide solutions for wine production in China.

## 2. Materials and Methods

### 2.1. LAB Strain and Growth Conditions

*L. hilgardii* Q19, isolated from the natural MLF process of Cabernet Sauvignon wines from the Qingtongxia appellation at the eastern foothills of Helan Mountain in China, was selected and preserved by our laboratory; Oeno1 (*O. oeni*) imported from France was the control strain.

The LAB strain was routinely grown in MRS medium (10.0 g/L peptone, 8.0 g/L beef extract powder, 4.0 g/L yeast extract powder, 2.0 g/L potassium phosphate dibasic, 2.0 g/L ammonium citrate dibasic, 5.0 g/L sodium acetate anhydrous, 0.2 g/L manganese sulfate, 0.04 g/L magnesium sulfate, and 1.0 mL/L Tween-80) at 28 °C for 24 h, incubated twice for purification, and incubated 28 °C for two days with 14 g/L agar in a CO_2_ incubator and maintained at −80 °C in glycerol (70% *v*/*v* final concentration). 

### 2.2. Bacterial Collection

The purified bacterial broth was strewed on an MRS Solid plate in three zones and cultured at 28 °C for 48 h. Typical single colonies were selected and inoculated in fresh MRS liquid medium for 19 h and used as seed liquid. The samples were inoculated at 2% of the inoculum in 5 L California vials and cultured at 28 °C for 36 h.

According to Li [17], the culture medium was centrifuged at 6000 rpm for 10 min at 4 °C to pellet microbial cells; the supernatant was discarded to collect the bacterial biomass. According to Xu et al. [18], the needed protective agent was weighed in advance, irradiated under ultraviolet for more than half an hour, and dissolved with sterilized sterile water.

### 2.3. Vacuum Freeze-Drying

The microbial suspension was prepared by mixing protective agents with each microbial strain in a 2:1 ratio. This suspension was stored in a refrigerator at 4 °C for 2 h for pre-freezing, producing cold-stress proteins to improve survival [19,20]. The microbial suspension was then placed in a freeze-drying dish with a thickness of 1.5 ± 0.1 cm and later freeze-dried using a vacuum freeze-dryer. The bacteria suspension was pre-frozen at −45 °C for 4 h to ensure that the suspension was in a solid state, and then the temperature was increased. The vacuum was pumped for sublimation drying and resolution drying to remove the free water and bound water in the suspension. The heating rate was maintained at 5 °C/min during this process to reduce the damage to the suspension caused by the formation of ice crystals, and the vacuum level at the sublimation stage was maintained at 0.2–0.4 mbar to improve the efficiency of sublimation drying [21,22]. Until the surface of the bacterium cake cracked, after which it was squeezed to form a powder, to gain lyophilized bacterium powder, this process needed to be kept sterile. Thus, a lyophilizer was used under UV conditions for more than 30 min after wiping with 75% alcohol before use.

### 2.4. Microbial Cell Count

The freeze-dried starter culture was redissolved in sterile water according to the original volume and kept at room temperature for 20 min. Then, a suitable gradient of the diluted bacterial solution was spread on culture media and incubated at 28 °C for 48 h. Finally, we counted the plates after they had grown bacteria. The experiment was repeated twice with three parallel sets for each experiment. The freeze-dried survival rate of *L. hilgardii* Q19 was calculated using the following formula:

survival rate/% = (N_f_/N_0_) × 100 (1)

Note: N_f_ represents bacterial survival after freeze-drying (CFU/mL), and N_0_ represents bacterial survival before freeze-drying (CFU/mL).

### 2.5. Single-Factor Experiment

Based on previous studies [17,18], protective agents were determined. We set up two levels to explore seven protectants for Q19 survival rate in the process of vacuum freeze-drying: 10, 15 g/100 mL skimmed milk powder; 10, 15 g/100 mL D-trehalose dehydrates; 10, 15 g/100 mL sucrose; 10, 15 g/100 mL yeast extract powder; 3, 4.5 g/100 mL glycerol; 5, 7.5 g/100 mL L-Glutamic acid sodium salt; 3, 4.5 g/100 mL D-sorbitol. Three protective agents with better protective effects were screened using sterile water as control group CK.

The optimal concentration screening was performed for the screened skimmed milk powder and yeast extract powder concentrations and were set to 0, 5, 10, 15, and 20 g/100 mL. L-Glutamic acid sodium salt concentrations were set to 0, 5, 7.5, 10 and 12.5 g/100 mL. The central point of the response surface was determined on this basis, and the experiment was repeated three times during the freeze-drying process.

### 2.6. Response Surface Optimization Experimental Design

Based on the results of the single-factor experiment, the contents of skimmed milk powder, yeast extract powder, and L-Glutamic acid sodium salt were selected as independent variables, and the freeze-drying survival rate of *L. hilgardii* Q19 was taken as the response value. The Box–Behnken Design method in Design-Expert.V.8 was used to conduct response surface analysis. The experimental factors and levels are shown in Table 1.

### 2.7. Low-Temperature and High-Alcohol Resistance in Pilot-Scale Fermentation Experiment

In order to explore the applicability of *L. hilgardii* Q19 starter in the actual production of wine, the starter was inoculated to the end of AF, and the wine without MLF was subjected to pilot-scale and low-temperature- and high-alcohol-resistant pilot-scaled fermentation experiments. The fundamental indexes of wine used for MLF are listed in Table 2.

Fermentation agent: Q19 starter culture; viable bacteria count was (4.36 ± 0.34) × 10^11^ CFU/g. Oeno1 (*O. oeni*) was used as the control agent with a viable count of (2.13 ± 0.19) × 10^11^ CFU/g.

Fermentation conditions: Q19 starter and Oeno1 starter were inoculated in the fermenter at 1 g/100 L, respectively, and the temperature was controlled for MLF.

MLF: Samples were taken every four days during the MLF process to detect the change in L-malic acid content. When the content reached 0.1 g/L, it was regarded as the end of fermentation. Potassium sulfite was added at a concentration of 60 mg/L to terminate the fermentation, and the contents of volatile components and biogenic amines were measured before and after fermentation.

### 2.8. Analytical Determinations

#### 2.8.1. Viable Count

The number of viable bacteria was determined according to GB 4789.35-2016 National Standard for Food Safety Microbiology Test of LAB [23]. The method is consistent with 2.4.

#### 2.8.2. L-Malic Acid Determination

Wines without MLF were used for the MLF assay. After filtration with a 0.22 µm membrane, 1 g/100 L of starters were inoculated into the wines. Wines without MLF were used as controls. Samples were collected every four days to determine changes in L-malic acid content. L-malic acid was measured enzymatically with Analyzer Y15 (Biosystems, Food Quality, Barcelona, Spain).

#### 2.8.3. Volatile Composition Analysis

Volatile components were determined by referring to the method of Bai [10] for GC−MS instrumentation. The volatile compounds of wines were identified using gas chromatography–mass spectrometry (HS-SPME-GC-MS) (Agilent 7890B gas chromatography in tandem with an Agilent 7000D mass spectrometer) (Agilent Technologies, Santa Clara, CA, USA) with an autosampler system (PALRSI 85) (CTC Analytics AG, Zwingen, Switzerland). The separation was performed on a DB-WAX capillary column (30 m × 0.25 mm × 0.25 µm, Agilent, USA). Next, 5 mL of wine, 1.5 g NaCl, and 10 µL 4-methyl-2-pentanol (2.01 mg/L) were placed into a 20 mL headspace vial, and the headspace vial was placed in the autosampler. The temperature was kept at 45 °C for 5 min, followed by extraction at 45 °C for 35 min. The injection temperature was 230 °C, and the analysis was carried out for 8 min. The injection mode was splitless injection. The carrier gas was high-purity helium and the flow rate was 1 mL/min. The GC-MS temperature program was as follows: An initial temperature of 40 °C was maintained for 5 min and increased by 3 °C/min to 130 °C/min, followed by an increase to 144 °C at a rate of 2 °C/min, then 5 °C/min to 240 °C, and then held for 10 min. The MSD transfer line heater was set to 240 °C. The temperature of the ion source was 230 °C. The mass detector was operated in full scan mode (*m*/*z* 40–300) with electron ionization (EI) mode at 70 eV. All compounds were analyzed using the NIST08 standard mass spectral library. 4-methyl-2-pentanol was used as an internal standard to calculate the relative content of compounds.

#### 2.8.4. Biogenic Amine Test

According to the National Standard for Food Safety: Determination of Bioamines in Food (GB/T 5009.208-2016), biogenic amine content in wine before and after MLF was determined [24].

### 2.9. Statistical Analysis

The data were analyzed statistically by one-way analysis of variance (ANOVA) with SPSS Statistics 22 (Chicago, USA), and the LSD method was compared afterwards. Duncan’s multiple range test compared means. Differences with *p* values <0.05 were considered statistically significant. Design-Expert.V.8 software (Beijing, China) was used for response surface analysis, and GraphPad Prism 9 and Origin 2021 software were used for mapping.

## 3. Results

### 3.1. Selection of the Optimal Concentration of Protective Agents

This research used seven protective agents (skimmed milk powder, yeast extract powder, D-trehalose dihydrate, D-sorbitol, glycerol, sucrose, and L-Glutamic acid sodium salt) were set at two levels, and the protective effects of lyophilization were investigated by single-factor experiment. The high level was 1.5 times that of the low level. Sterile water was used as the control group. As shown in Table 3, the order of the protective effects of the seven protective agents on *L. hilgardii* Q19 was skimmed milk powder > L-Glutamic acid sodium salt > yeast extract powder > sucrose > D-trehalose dehydrate > D-sorbitol > glycerol > sterile water. The protective agent could provide physical support for bacteria and reduce biochemical damage caused by the freeze-drying process. Sterile water without a protective agent showed a low freeze-drying survival rate, so adding a protective agent significantly affected the freeze-drying survival rate of Q19. Among them, skimmed milk powder, yeast extract powder, and L-Glutamic acid sodium salt had the most apparent protective effect: the lyophilized survival rate of these three protective agents was above 60% at both high and low levels of concentration, so these three protective agents were selected for further analysis.

There are different enumeration methods for viable LAB cells and the freeze-drying survival rate. Skimmed milk powder is a kind of polymer compound protective agent that can provide an excellent protective effect when used alone or separately [25]. The protective mechanism can be explained by the fact that during freeze-drying, whey protein can wrap around the surface of bacterial cells, protect the cell membrane, and fix freeze-drying enzymes [26]. The L-Glutamic acid sodium salt is an antioxidant for starters during storage. The mechanism of protection for lactic acid bacteria is the reaction between the amino group of sodium glutamate and the carboxyl group of the cell protein of the bacterium, which stabilizes the structure of the cell protein of the microorganism. It can retain more residual water to maintain cell-life activity [27]. Yeast extract powder was less involved in previous freeze-drying experiments. This study found that during vacuum freeze-drying, the surface of the cake with yeast immersion powder as the protective agent was easy to crack, which was conducive to the evaporation of the internal water of the bacterial solution. The yeast immersion powder also made it easy to freeze-dry the bacterial solution and maintained a high survival rate after freeze-drying. In addition, since the yeast extract powder was added to the MRS medium for Q19 culture and re-added as a protective agent before freeze-drying, both could adapt well and play a better protective role. This is similar to the study of Tymczyszyn et al. [28], who added sucrose into MRS medium when cultivating lactic acid bacteria to improve the protective effect of sucrose. 

In conclusion, skimmed milk powder, yeast extract powder, and L-Glutamic acid sodium salt were selected for further single-factor experiments, and the central point of the response surface was selected.

### 3.2. Response Surface Optimization

#### 3.2.1. The Response Surface 

**Figure 1:**
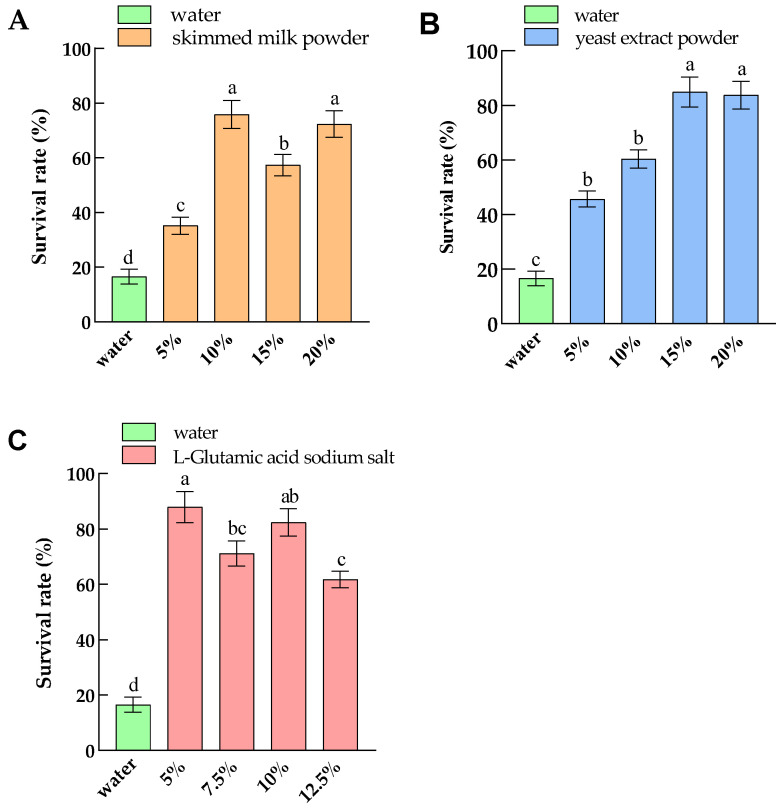
The effect of protective agent dosage on the vitality of freeze-dried Q19. (**A**) skimmed milk powder; (**B**) yeast extract powder; (**C**) L-Glutamic acid sodium salt. a, b and c indicate significance analysis.

Through single-factor experiment, the effects of different concentrations of skimmed milk powder, yeast extract powder, and L-Glutamic acid sodium salt on the survival rate of lyophilized *L. hilgardii* Q19 were investigated, and the optimal concentrations of the three protective agents were determined. As shown in Figure 1, the three protective agents had significant effects on the freeze-drying survival rate of Q19 (*p* < 0.05). The maximum freeze-drying survival rate of *L. hilgardii* Q19 appeared when the amount of skimmed milk powder was 10 g/100 mL. The lyophilized survival rate of Q19 remained above 60% during the change of L-Glutamic acid sodium salt addition between 5, 7.5, 10, and 12.5 g/100 mL, indicating that L-Glutamic acid sodium salt had an excellent protective effect on the vacuum freeze-drying of Q19, which is consistent with the study of Dimitra et al. [21]. When the supplemental level of L-Glutamic acid sodium salt was 5 g/100 mL, the freeze-drying survival rate of Q19 reached the maximum; there was no statistically significant difference compared with 10 g/100 mL. The freeze-drying survival rate of Q19 increased with the dosage of yeast extract powder, and the maximum value was found when the dosage of yeast extract powder was 15 g/100 mL.

According to comprehensive analysis, the content of Q19 in bacterial powder per unit mass decreased due to the increase in protective agent dosage. Therefore, considering the total cost and bacterial powder activity, the additional levels of skimmed milk powder, L-Glutamic acid sodium salt, and yeast extract powder were determined to be 10, 5, and 15 g/100 mL, respectively, and this was taken as the central point of the response surface for response surface analysis.

#### 3.2.2. Response Surface Experiment and Result Analysis

The result of the Box–Behnken experiment showed in Table 4. A response surface method experiment with three factors and three levels with 17 experimental points was selected, in which the number of experiments of the factorial part was 12 and the number of experiments of the central point was 5 to estimate the experimental variance. The regression equation fitted to the experimental results according to Design-Expert.V8.1 was Y = 85.78 − 1.12A − 0.52B + 3.72C + 0.7AB − 3.62ac − 0.012BC − 2.85A2 − 2.11B2 − 4.27C2, and the results of variance analysis are shown in Table 5.

Table 5 showed that the model established in this experiment was highly significant (*p* < 0.01); the misfit term was not significant (*p* > 0.05), so the reliability of the model was high. From the primary term of the F-value, it can be seen that skimmed milk powder, yeast extract powder, and L-Glutamic acid sodium salt had significant effects on the lyophilization survival rate of Q19, with L-glutamate having a highly significant effect. From the F-value interaction term, the effect on the lyophilization survival rate of Q19 was highly significant when skimmed milk powder and L-Glutamic acid sodium salt were combined. Furthermore, R^2^ = 0.9759 indicated that the fit between the predicted and true values was good. The lower coefficient of variation (CV) indicates the higher reliability of the experiment, and the coefficient of variation in the test was 1.31%, proving that the experimental results were credible. Collectively, the model can target the lyophilization survival rate of Q19 for prediction.

**Figure 2:**
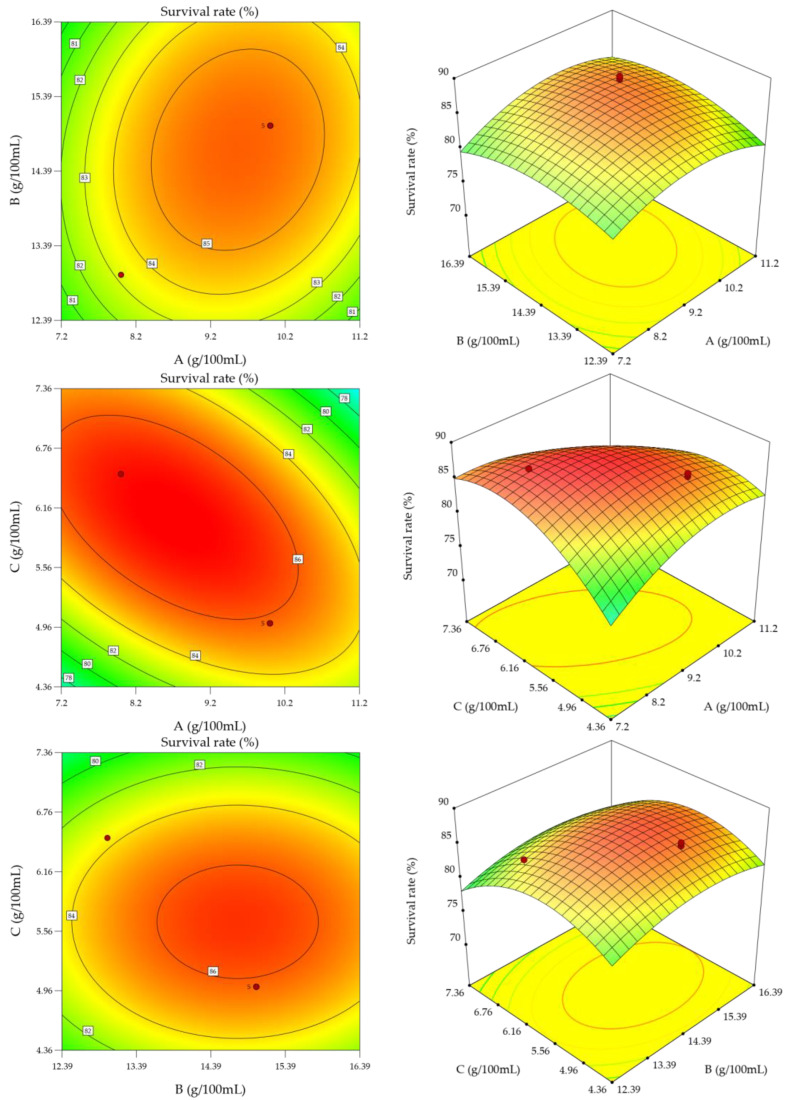
Response surface and contour of the interaction of various factors. Red dots indicate the maximum survival rate of two variables, and the numbers in the images indicate the survival rate values.

Response surface plots and contour plots can show the interaction between two factors; steeper response surfaces indicate that the interaction between the two factors has a greater degree of influence on the response value, and contour line that are closer to the ellipse indicate that the interaction between the two factors is more significant [29]. The response surface and contours obtained from the experiment are shown in Figure 2. When the L-Glutamic acid sodium salt was kept constant at 5 g/100 mL, the lyophilized survival rate of Q19 showed a trend of increasing and then decreasing as the amount of skimmed milk powder and yeast extract powder was increased. When the amount of skimmed milk powder was between 8.42 and 10.7 g/100 mL, and the amount of yeast extract powder was between 13.35 and 16 g/100 mL, the contours showed a closed curve, indicating a maximum value in this interval. When the amount of yeast extract powder was kept constant at 15 g/100 mL, the lyophilization survival rate of Q19 showed a trend of increasing and then decreasing with the amount of skimmed milk powder and L-Glutamic acid sodium salt. The optimal value occurred when the amount of skimmed milk powder was 8.0–10.9 g/100 mL and L-Glutamic acid sodium salt was 4.7–6.5 g/100 mL. The contour lines showed an elliptical shape, and the steep response surface indicated the significant interaction between skimmed milk powder and L-Glutamic acid sodium salt. When the amount of skimmed milk powder was held constant at 10 g/100 mL, the lyophilized survival rate of Q19 increased and then decreased with the addition of yeast extract powder and monosodium L-Glutamic acid sodium salt, with optimal values appearing in the contour closures of 13.04–16.5 g/100 mL for yeast extract powder and 4.72–6.5 g/100 mL for monosodium L-Glutamic acid sodium salt. The analysis results in Figure 2 were consistent with the significance in Table 5.

### 3.3. Validation Test

The results were brought into the model for statistical analysis using Design-Expert.V8 software, and the maximum survival rate occurred when 8.62 g/100 mL skimmed milk powder, 14.51 g/100 mL yeast extract powder, and 6.09 g/100 mL L-Glutamic acid sodium salt were used as the protective agent formulation. The predicted survival rate of lyophilized Q19 in this case was 87.58%. Three lyophilization replicate experiments were carried out to obtain the actual lyophilization survival rate of Q19 as 87.85 ± 1.19%, and the obtained results were close to the predicted values. The protective agent formulation was optimized to 8.5 g/100 mL skimmed milk powder, 14.5 g/100 mL yeast extract powder, and 6 g/100 mL L-Glutamic acid sodium salt. The results illustrated the reliability of the experimental results: in this case, the number of live bacteria in the Q19 starter culture was (4.36 ± 0.34) × 10^11^ CFU/g.

### 3.4. Q19 Starter Storage Performance

The ability of starter cultures to be stored for an extended period is a prerequisite for commercialization. Table 6 shows the change of viable LAB cells of the Q19 starter during storage. At storage temperatures of 4 °C and −20 °C, the number of viable LAB cells shows a decreasing trend with the increase in storage time. Among them, the number of viable bacteria decreased slowly at −20 °C, and the survival rate remained above 70% after 180 d of storage, which is consistent with the findings of Ren et al. [30]. This showed that the Q19 starter could maintain good activity at −20 °C.

### 3.5. Pilot-Scale MLF

#### 3.5.1. L-Malic Acid Conversion Rate during MLF

**Figure 3:**
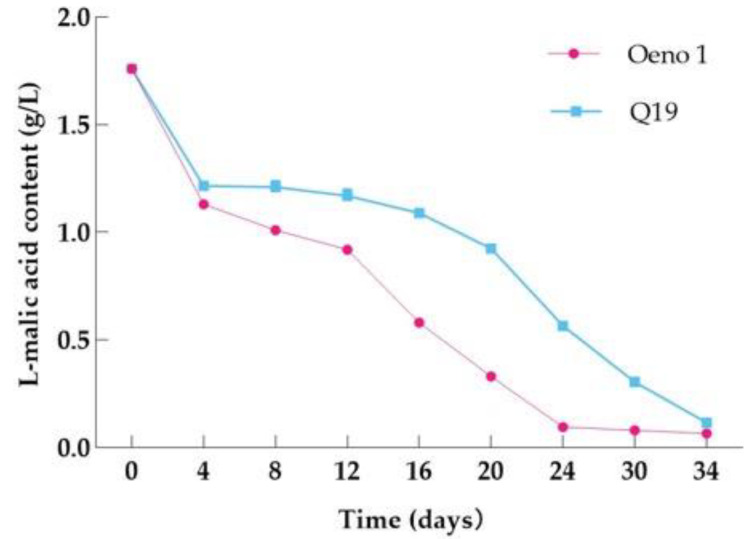
Changes of L-malic acid during MLF.

L-malic acid content changes during MLF are a crucial sign of whether or not MLF has begun. L-malic acid was examined in the process of MLF of Cabernet Franc and Yan 73 to evaluate the degradation rate of L-malic acid by two lactic acid fermenters, Q19 and Oeno1, and the results are shown in Figure 3. Along with the access of Q19 and Oeno1, the L-malic acid content showed a significant decreasing trend, indicating that the accessed Q19 and Oeno1 played a major MLF role in the MLF process. In addition, wine samples inoculated with Oeno1 completed MLF after 24 d, and those inoculated with Q19 completed MLF after 34 d. Oeno1 was able to complete MLF faster compared to Q19.

#### 3.5.2. Volatile Components Analysis

The volatile components produced by Q19 and control were analyzed after MLF, and the results showed in Table A1. Forty-nine volatile substances were detected in the wine samples without MLF, totaling 7.4693 mg/L. Inoculated with Q19, 66 volatile aroma substances were detected after MLF, including 15 alcohols, 27 esters, 14 acids, 5 terpenes, 3 aldehydes and ketones, and 2 others, with a total content of 9.8493 mg/L. In the wine samples inoculated with control, 55 volatile aroma substances were detected after MLF, including 14 alcohols, 21 esters, 10 acids, 4 terpenes, 2 aldehydes and ketones, and 4 others, with a total content of 7.2088 mg/L. Compared to non-MLF, inoculation of LAB with MLF enhanced the type and content of terpenoids; the wine samples inoculated with Q19 produced more types and content of esters, acids, and terpenoids compared to the control. 

The alcohol concentration was 1.101 mg/L in the wines that were inoculated with Q19 after MLF, higher than the control. Alcohols are mainly produced from the metabolism of amino acids during AF. Higher alcohols can significantly contribute to a wine’s aromatic profile, adding fruit and floral aromas and aromatic complexity. However, when the content of higher alcohols is higher than 400 mg/L, they can produce a pungent and unpleasant aromatic profile that can negatively affect the wine aroma [31]. According to Table A1, the type and concentration of esters in the wine samples inoculated with Q19 were significantly higher than those in control. In contrast to the control, the wine samples inoculated with Q19 produced isoamyl decanoate, ethyl myristate, and butyrolactone, which gave the wine distinct aromas like rose, cinnamon, butter, and caramel. Esters have a more significant impact on wine aroma than alcohols and usually impart floral and fruity aromas to wines. However, when their contents are too high, they can mask the varietal aromas and reduce the aromatic complexity of the wine, and their concentration decreases during aging due to chemical hydrolysis.

#### 3.5.3. PCA Analysis of Different Fermentation Starter

**Figure 4:**
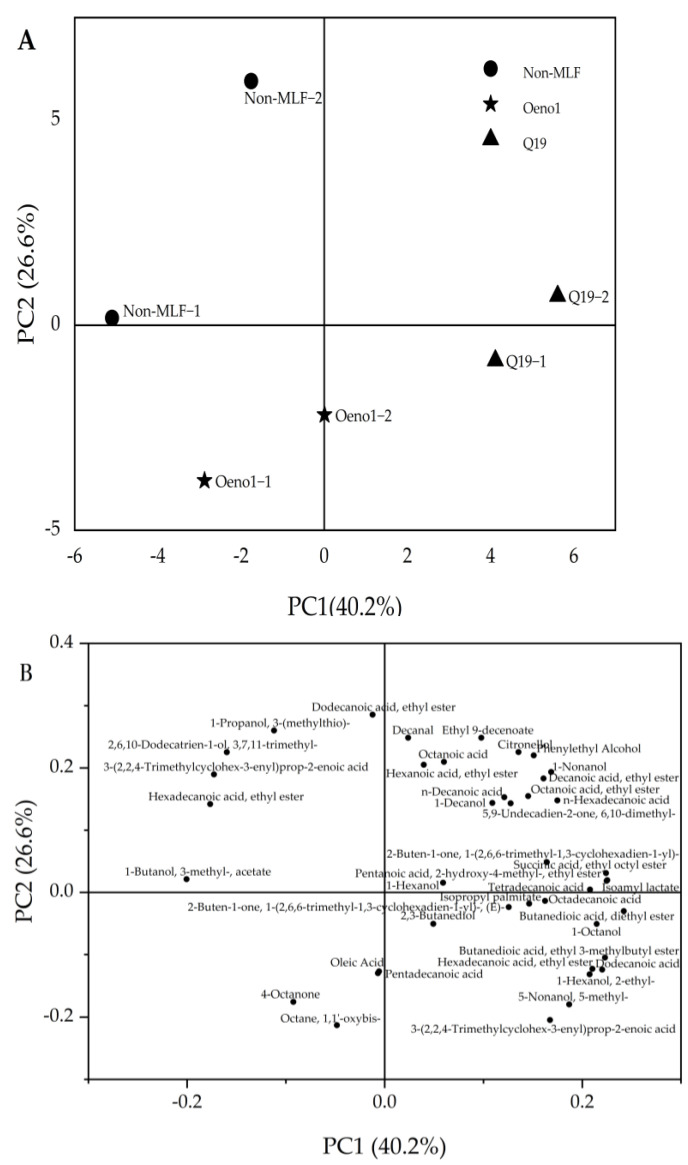
Principal component analysis of aroma compounds in fermented wine samples. Score plot (**A**) and loading plot (**B**).

In order to better distinguish the differences in volatile components between control and Q19 after MLF, principal component analysis (PCA) analysis was performed on all 41 volatile aroma components with volatile components greater than 10 μg/L using Origin 2021 software. The obtained score plot (A) and loadings plot (B) are shown in Figure 4. The cumulative variance contribution of the two extracted principal components, PC1 (40.2%) and PC2 (26.6%), was 66.8%. The experimental groups had different distributions in Figure A due to the different inoculation of starters. The distribution of wine samples inoculated with Q19 was mainly in the positive direction of PC1, whereas the distribution of wine samples inoculated with Oeno1 was predominantly in the negative direction of PC1. The upper right of Figure B had the most volatile aroma substances, including six esters, six acids, four alcohols, and three terpenes, which give the wine its unique floral, fruity, honey, and other characteristic aromas associated with the fermentation of Q19 in Figure A. The oleic acid, pentadecanoic acid, 4-octanone, and n-noctyl ether in the lower left part of Figure B correlated with the Oeno1 fermentation in Figure A. Collectively, the wine samples inoculated with Q19 produced more volatile aroma substances than the control.

#### 3.5.4. Wine Safety 

To evaluate the safety of the wine samples after Q19 inoculation, the concentration of eight biogenic amines was evaluated in the wine samples before and after fermentation; the findings are shown in Table 7. It can be seen that β-phenylethylamine, putrescine, histamine, and tyramine contents were reduced in the wine samples inoculated with Q19 compared to the control. The histamine content was 6.38 mg/L, which was lower than the limits for histamine content in France, Australia, and other countries [32]. In addition, wine samples inoculated with Q19 produced spermine after MLF, but the total biogenic amine content was less than the control. On balance, the Q19 fermenter produced fewer biogenic amines and was more in line with the current demand for “green” LAB starters.

### 3.6. Analysis of Fermentation Performance in a Pilot Test for Low-Temperature and High-Alcohol Resistance

#### 3.6.1. Bacterial Density Variation during MLF

**Figure 5:**
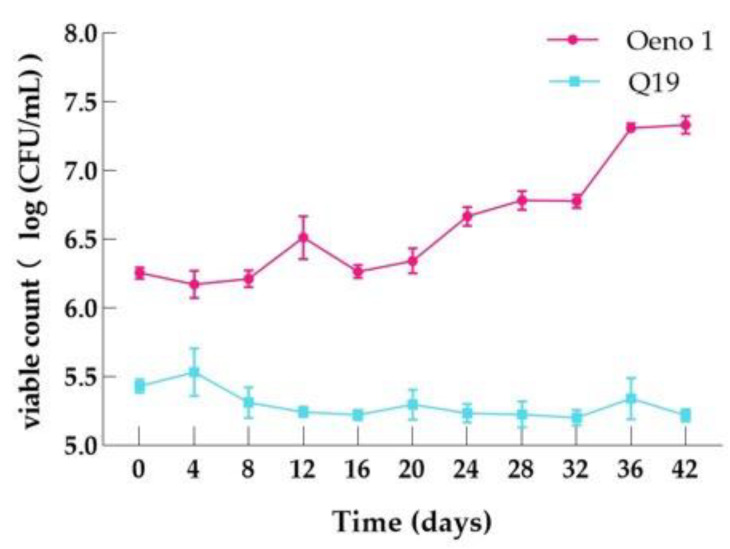
Changes in bacterial density during MLF.

As shown in Figure 5, inoculation with both Q19 and control resulted in good survival in the wine environment, with no significant order-of-magnitude decrease. Among them, Q19 maintained a live count of 10^5^ CFU/mL throughout the MLF process. The wine samples inoculated with control maintained a stable live count of 10^6^ CFU/mL from 0 to 20 days of fermentation. From 20 to 36 days, the viable control count showed a gradual increase, with the viable count growing from 10^6^ CFU/mL to 10^7^ CFU/mL; at 36 days to the end of MLF, the viable count of the control began to stabilize and did not show an increasing trend.

#### 3.6.2. L-Malic Acid Changes during MLF

**Figure 6:**
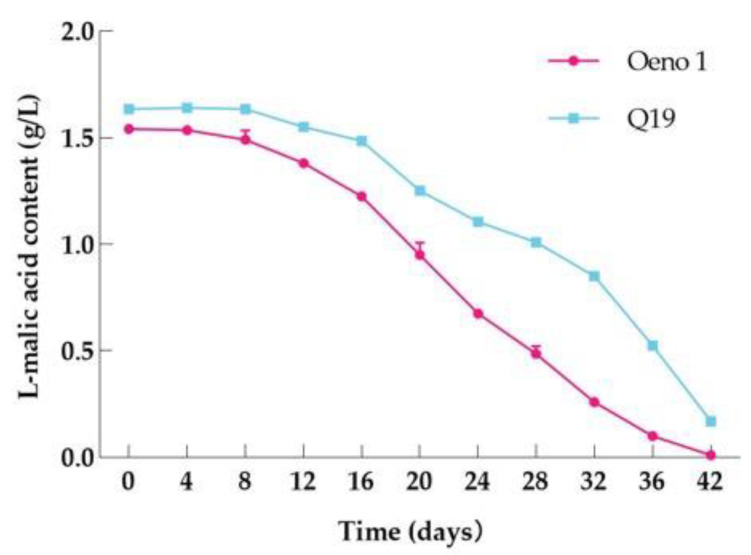
Changes of L-malic acid during MLF.

As shown in Figure 6, L-malic acid showed a slow decline followed by a linear decline after LAB inoculation, and there were some differences in the L-malic acid degradation rate between Q19 and the control rate. The comprehensive analysis showed that the MLF of Q19 started more slowly, and the MLF cycle was longer than the control.

#### 3.6.3. Analysis of Volatile Aroma Components in Wine

The volatile components of each wine sample were measured before and after MLF fermentation to compare and evaluate the effects of the two starters on the wine aroma substances. The results are shown in Table A2: 45 volatile compounds were detected before MLF with a total content of 3.6826 mg/L. A total of 49 volatile compounds were detected in the wine samples inoculated with Q19 after MLF, with a total content of 4.304 mg/L, including 12 alcohols, 21 esters, 8 acids, 3 terpenoids, and 3 hydroxyl compounds. A total of 47 volatile compounds were detected in wine samples inoculated with control, with a total content of 4.9642 mg/L, including 14 alcohols, 20 esters, 5 acids, 3 terpenes, and 3 aldehydes and ketones. After MLF, there was a significant increase in the type and content of esters, with Q19 producing a greater variety of esters, but the control produced a greater content of esters.

#### 3.6.4. Wine Safety

Performing MLF at low temperatures results in longer cycles and may cause elevated biogenic amine levels. As shown in Table 8, five biogenic amines, tryptamine, β-phenylethylamine, cadaverine, histamine, and tyramine were detected before and after MLF. After the completion of MLF, the content of biogenic amines in wine samples inoculated with Q19 was 33.34 mg/L and that of wine samples inoculated with control was 40.17 mg/L. Compared with the non-MLF, the content of cadaveric amines, histamine, and tyramine in wine samples inoculated with Q19 showed different degrees of decrease, and the total biogenic amine content decreased by 2.32 mg/L. The wine samples inoculated with control showed different degrees of increase in tryptamine, β- phenylethylamine, and tyramine, and the total biogenic amine content increased by 4.51 mg/L. In addition, no cadaverine, which has a high impact on health, was detected in the wine samples inoculated with Q19.

## 4. Discussion

The preparation of DVS starter cultures provides the conditions for the industrial application of desirable strains. However, there are few studies on MLF starters in China. Cheng et al. [33] used 10% skimmed milk powder, 13% sucrose, 2% sorbitol, and 0.8% tyrosine as freeze-drying protectants in the vacuum freeze-drying of *L. plantarum* L1, and the survival rate reached 97.4% after freeze-drying. The study showed that lyophilized milk powder, as a better protective agent, should be used in combination with other protective agents to achieve better protection. In this study, Q19 DVS starter was prepared using *L. hilgardii* Q19 with a formulation of 8.5 g/100 mL skimmed milk powder, 14.5 g/mL yeast extract powder, and 6.0 g/100 mL L-Glutamic acid sodium salt as the lyophilization protectant. The survival rate was 87.85% under these conditions, and the number of viable LAB cells reached 4.36 × 10^11^ CFU/g. During the lyophilization process, it was found that higher or lower dosages of protective agents were not ideal for the protection of the bacteria, which was similar to the results of previous studies [34,35], in which both high osmotic pressure and rapid changes in osmotic pressure disrupted the cell membrane of the bacteria, thus reducing the survival rate. Other studies showed that the strain, growth conditions, freeze-drying technology, and protective effect of different protectants are the primary determinants of the survival rate of lyophilized strains [36]. In addition to the lyophilization protectants investigated in this work, the anti-freezing ability of the bacteria is important for the generation and preservation of highly active starters [37].

Seong Choi et al. [38] found that with the cold treatment of *Lactobacillus brevis* (*L. brevis*) WiKim0069 before vacuum freeze-drying, the expression of potential frozen surface layer protein (SLP) was promoted, increasing the storage time and survival rate of *L. brevis* WiKim0069 after freeze-drying. The ideal cold-treatment temperature of *L. hilgardii* Q19 before vacuum freeze-drying is a topic for future research to improve the survival and storage time of *L. hilgardii* Q19 after freeze-drying. In this study, the survival rate of *L. hilgardii* Q19 was 87.85% after freeze-drying, which may be related to anhydrobiosis. Related studies found that by rehydrating, anhydrobiosis can increase cellular energy generation and increase the capacity of self-repair in a hostile environment [39,40]. Wang et al. [41] found no significant change in survival with storage time when the starter was frozen and preserved at −80°C. This indicated that the −80°C environment is appropriate for preserving ferments and might be studied for future starter storage.

After MLF, the metabolic activity of LAB results in fuller-bodied wines with more intense flavors; this change shows the complexity of LAB in the metabolic process of wine [42]. Another study showed that *Lactobacillus* is more tolerant to the microbial wine environment. That *Lactobacillus* has more wine aroma-related enzyme genes than *O. oeni* and produces bacteriocins—which would enable them to combat LAB spoilage—implies that bacteria of the genus *Lactobacillus* have excellent potential for the development and application of future MLF starters [43,44]. During the pilot-scale MLF, the conversion rate of L-malic acid control was faster than that of Q19, which may be because *O. oeni* tends to be more tolerant to the wine-fermentation environment [45]. Other studies showed that *O. oeni* can form biofilms during MLF, and these biofilms can effectively compose MLF-tolerant cells and successfully complete MLF [46]. In the pilot-scale fermentation with low-temperature and high-alcohol-tolerance MLF, the growth of viable LAB cells in Q19 was slower than the control, and there was no significant order of magnitude increase. This is consistent with the finding of Zhang et al. [47] that suitable environmental conditions can significantly enhance the productivity of *O. oeni* cultures. The MLF time of Q19 was longer compared to the control, may be related to chaotropicity-mediated ethanol stress. Santos et al. [48] found that mycobacterium may have the highest ethanol tolerance so far reported for cells of bacteria or Archaea. In addition, some studies showed that chaotropic solute has an inhibitory effect on water bodies, soils, and biofuel-producing microorganisms [49,50]. Hallsworth et al. [51] found that ethanol-induced water stress eventually disrupted enzyme and membrane structure and function. Ethanol is mildly chaotropic at low concentrations and can reduce the stability of macromolecules [52]. The stress induced by ethanol can be counteracted by absorption and/or synthesis of compatible solutes (e.g., trehalose, glutamate), which can stabilize the macromolecular systems [53,54,55]. In addition, ethanol inhibits microbial metabolism by reducing water activity and causing stress [56,57]. Therefore, we speculated that it was related to no significant increase in the viable count of Q19 in the pilot-scale experiment. We will carry out more pilot-scale fermentation experiments in the future and discuss the results. 

In summary, *L. hilgardii* Q19 was tolerant to various stresses in wine-fermentation environments. This enhanced its vitality, making it adaptable and competitive between species in a survival environment [58,59]. Terpenoids and esters play an important role in regulating wine aromas [60]. Yeast and LAB can produce esters and terpenes during winemaking, during which LAB release volatile glycosides from glucoside precursors to increase the terpene content of the wine [61,62]. In a comparative study of the winemaking characteristics of *L. plantarum* CX19 and *O. oeni* PN4 by Sun et al. [63], *L. plantarum* CX19 was more tolerant of high pH environments than *O. oeni* PN4, and wines fermented with *L. plantarum* CX19 had a more complex aroma composition and better taste. In this study, the pilot-scale fermentation revealed that after MLF, Q19 produced more types and levels of esters and terpenes compared to the control, indicating that the aroma composition of wine samples inoculated with Q19 was more intense and complex, similar to the results of the above study. Inoculated wine samples with Q19 produced unique volatile aroma substances such as butyrolactone, isobutyl n-octanoate, ethyl octyl succinate, and damascenone, which gave the wines floral and fruity aromas and preserved terroir characteristics while retaining indigenous microbial diversity. In the pilot-scale fermentation at low temperature and high alcoholic strength, the ester content of the fermented wine samples were all elevated after fermentation compared to non-MLF, in agreement with the finding of Iorizzo et al. [61]. Compared to the control, the wine samples inoculated with Q19 produced more terpenoids, which may be because *Lactobacillus* spp. strains carry more coenzyme genes related to aroma [43,44].

Another essential feature of MLF’s LAB starter is that it does not produce biogenic amines during the winemaking process, which impacts the wine’s safety; some biogenic amines (e.g., putrescine) can also affect the aroma of the wine [47]. Patrignani et al. [64] studied the concentration of biogenic amines in 160 bottles of Italian red wine, in which tyramine concentrations ranged from 1.58 to 10.19 mg/L and histamine concentrations ranged from 1.49 to 16.34 mg/L. Li et al. [65] studied the concentration of biogenic amines in 39 Chinese red wines and found that the maximum values of histamine, tyramine, and β-phenylethylamine were 10.51, 9.31, and 4.58 mg/L, respectively. While there are no clear limits on the acceptable amount of biogenic amines in wine in the EU, in this study, the pilot-scale fermentation wine samples inoculated with Q19 after MLF produced reduced levels of β-phenylethylamine, putrescine, histamine, and tyramine and produced less total biogenic amines compared to the control. In particular, histamine and tyramine, which have a major impact on human health, had contents of 6.38 and 2.1 mg/L, respectively, in the wine samples inoculated with Q19, which meet the current evaluation criteria for healthy wines.

## 5. Conclusions

In this study, the optimal formulation of *L. hilgardii* Q19 freeze-dried lyoprotectants was determined to be 8.5 g/100 mL skimmed milk powder, 14.5 g/100 mL yeast extract powder, and 6.0 g/100 mL L-Glutamic acid sodium salt, yielding a freeze-dried survival rate of 87.85 ± 1.19% and a live count of Q19 starter of 4.36 ± 0.34 × 10^11^ CFU/g. Q19 starter degrades L-malic acid well in real production to suit the demands of wine MLF, and it can complete MLF under low-temperature and high-alcohol circumstances, solving the difficulties of low temperatures and high alcohol contents faced by wine MLF in China and some other production locations. After MLF, the wine samples inoculated with Q19 produced more types and levels of volatile aromatic substances, especially esters and terpenes, than the commercial control, with volatile aromatic substances such as acacia alcohol and damascenone, giving the wine unique aromatic characteristics such as honey, rose, and floral notes. In addition, the wine samples inoculated with Q19 produced lower levels of biogenic amines compared to the control. This conclusion provides a basis for preparing highly active Q19 starter and its future production, but further research is needed for *L. hilgardii* to carry enzyme genes related to characteristic aromas.

## Figures and Tables

**Table 1:** Factors and levels of Box–Behnken tests for compounding of protective agents.

Level	Skimmed Milk Powder (A) /(g/100 mL)	Yeast Extract Powder (B) /(g/100 mL)	L-Glutamic Acid Sodium Salt (C)/(g/100 mL)
−1	8	13	3.5
0	10	15	5
+1	12	17	6.5

**Table 2:** Basic indexes of wine simples used in MLF.

Index	A Value	B Value
Reducing sugars (g/L)	1.13	18.11
Effective alcohol degree (% *v*/*v*)	12.93	14.6
Total acidity (g/L tartaric acid equivalent)	5.88	6.67
Volatile acidity (g/L acetic acid equivalent)	0.27	0.33
pH	3.84	3.53
Grape varietal	Cabernet Franc and Yan 73	Cabernet Sauvignon
Wine type	Dry Red Wines	Dry Red Wines
Scale (t)	5	1
Fermentation temperature (°C)	18 ± 2	15 ± 1

Note: A: pilot-scale, B: high-alcohol low-temperature pilot-scale.

**Table 3:** Effect of different protective agents on the survival rate of Q19 freeze-drying.

Lyoprotectants /(g/100 mL)	Before Freeze-Drying Viable LAB Cells /(CFU/mL)	After Freeze-Drying Viable LAB Cells /(CFU/mL)	Freeze-Drying Survival Rate /(%)
D-trehalose dihydrate 10	(4.46 ± 0.12) × 1010 ***	(1.92 ± 0.15) × 10^10^	43.28 ± 4.87 ^e^
D-trehalose dehydrate 15	(6.26 ± 0.47) × 1010 ***	(2.66 ± 0.20) × 10^10^	42.55 ± 0.29 ^e^
glycerol 3	(4.86 ± 0.15) × 1010 ***	(3.13 ± 0.25) × 10^9^	6.43 ± 0.67 ^g^
glycerol 4.5	(4.80 ± 0.20) × 1010 ***	(8.66 ± 0.12) × 10^9^	18.06 ± 3.78 ^f^
yeast extract powder 10	(5.20 ± 0.10) × 1010 ***	(3.26 ± 0.12) × 10^10^	62.82 ± 2.70 ^c^
yeast extract powder 15	(6.00 ± 0.46) × 1010 *	(4.26 ± 0.12) × 10^10^	71.11 ± 7.83 ^b^
skimmed milk powder 10	(6.00 ± 0.17) × 1010	(5.20 ± 0.26) × 10^10^	86.67 ± 6.94 ^a^
skimmed milk powder 15	(5.52 ± 0.25) × 1010 **	(4.20 ± 0.10) × 10^10^	75.90 ± 3.47 ^b^
D-sorbitol 3	(5.46 ± 0.32) × 1010 ***	(8.66 ± 0.06) × 10^9^	15.85 ± 0.40 ^f^
D-sorbitol 4.5	(6.06 ± 0.06) × 1010 ***	(1.06 ± 0.06) × 10^10^	17.58 ± 1.55 ^f^
L-Glutamic acid sodium salt 5	(5.12 ± 0.16) × 1010 ***	(4.00 ± 0.10) × 10^10^	77.92 ± 2.00 ^b^
L-Glutamic acid sodium salt 7.5	(5.92 ± 0.23) × 1010 ***	(3.80 ± 0.26) × 10^10^	64.04 ± 4.28 ^c^
sucrose 10	(4.60 ± 0.10) × 1010 ***	(2.40 ± 0.10) × 10^10^	52.17 ± 2.08 ^d^
sucrose 15	(2.60 ± 0.20) × 1010 ***	(1.40 ± 0.10) × 10^10^	53.85 ± 0.61 ^d^
sterile water	(3.06 ± 0.15) × 1010 ***	(5.06 ± 0.15) × 10^9^	16.52 ± 0.63 ^f^

Note: “*” indicates a significant effect on the results (*p* < 0.05); “**” indicates a highly significant effect on the results (*p* < 0.01); “***” indicates a highly significant effect on the results (*p* < 0.001). ^a^, ^b^, ^c^, ^d^, ^e^, ^f^ and ^g^ indicate significance analysis. The populations were tested before and after freeze-drying and tested within the column of the freeze-drying survival rate.

**Table 4:** Results of Box–Behnken tests.

Number	A/g/100 mL	B/g/100 mL	C/g/100 mL	Survival Rate/%
1	10.00	15.00	5.00	86.23
2	8.00	15.00	6.50	87.32
3	12.00	17.00	5.00	80.77
4	10.00	13.00	6.50	84.32
5	10.00	15.00	5.00	85.72
6	8.00	17.00	5.00	81.48
7	10.00	17.00	6.50	81.58
8	12.00	13.00	5.00	78.67
9	12.00	15.00	6.50	77.78
10	10.00	15.00	5.00	84.78
11	8.00	15.00	3.50	72.29
12	10.00	15.00	5.00	86.67
13	8.00	13.00	5.00	82.32
14	10.00	15.00	5.00	85.48
15	10.00	13.00	3.50	77.19
16	10.00	17.00	3.50	74.50
17	12.00	15.00	3.50	77.23

**Table 5:** Variance analysis of response surface methodology results.

Source	Sum of Squares	Degree of Freedom	Mean Square	F-Value	*p*-Value	Significant
Model	321.02	9	35.67	31.51	<0.0001	**
A	10.04	1	10.04	8.86	0.0206	*
B	2.17	1	2.17	1.92	0.2084	
C	110.93	1	110.93	97.99	<0.0001	**
AB	2.16	1	2.16	1.91	0.2096	
AC	52.42	1	52.42	46.30	0.0003	**
BC	6.250 × 10^−4^	1	6.250 × 10^−4^	5.521 × 10^−4^	0.9819	
A^2^	34.30	1	34.30	30.30	0.0009	**
B^2^	18.78	1	18.78	16.59	0.0047	**
C^2^	76.65	1	76.65	67.71	<0.0001	**
Residual	7.92	7	1.13			
Lack of Fit	5.84	3	1.95	3.73	0.1181	
Pure Error	2.09	4	0.52			
Cor Total	328.95	16				
R^2^ = 97.59				C.V.% = 1.31		

Note: “*” indicates a significant effect on the results (*p* < 0.05); “**” indicates a highly significant effect on the results (*p* < 0.01).

**Table 6:** Change in viable LAB cells during storage.

Temperature	0 Day /10^11^ CFU/g	7 Days /10^11^ CFU/g	30 Days /10^11^ CFU/g	90 Days /10^11^ CFU/g	180 Days /10^11^ CFU/g
4 °C	4.35 ± 0.54 ^a^	4.23 ± 0.34 ^ab^	4.01 ± 0.36 ^ab^	3.63 ± 0.45 ^ab^	3.23 ± 0.33 ^b^
−20 °C	4.35 ± 0.54 ^a^	4.21 ± 0.32 ^a^	4.09 ± 0.28 ^a^	3.71 ± 0.25 ^ab^	3.40 ± 0.26 ^b^

Note: ^a^ and ^b^ indicate significance analysis.

**Table 7:** Biogenic amine in Cabernet Franc and Yan 73 wines.

Group	Tryptamine mg/L	β-Phenylethylamine mg/L	Putrescine mg/L	Cadaverine mg/L	Histamine mg/L	Tyramine mg/L	Spermine mg/L	Total mg/L
Non-MLF	\	6.94 ± 0.29 ^a^	11.96 ± 1.89 ^b^	0.31 ± 0.05 ^c^	9.80 ± 0.11 ^a^	3.63 ± 0.38 ^ab^	ND	31.75 ± 2.00 ^b^
Oeno1	\	7.07 ± 0.92 ^a^	16.35 ± 2.51 ^a^	2.18 ± 0.00 ^b^	10.28 ± 1.26 ^a^	4.3 ± 0.18 ^a^	ND	39.53 ± 0.11 ^a^
Q19	\	4.15 ± 0.41 ^b^	11.12 ± 0.52 ^b^	4.00 ± 0.07 ^a^	6.38 ± 1.61 ^b^	2.1 ± 0.43 ^b^	6.49 ± 0.93 ^a^	33.42 ± 3.10 ^b^

Note: ^a^, ^b^ and ^c^ indicate significance analysis, and “ND” indicates that the biogenic amine component was not detected. Tested within column.

**Table 8:** Biogenic amine in Cabernet Sauvignon wines.

Group	Tryptamine mg/L	β-Phenylethylamine mg/L	Putrescine mg/L	Cadaverine mg/L	Histamine mg/L	Tyramine mg/L	Spermine mg/L	Total mg/L
Non-MLF	8.37 ± 0.12 ^b^	9.70 ± 0.85 ^b^	\	0.58 ± 0.27 ^a^	12.83 ± 3.76 ^a^	4.41 ± 0.81 ^ab^	\	35.66 ± 3.02 ^a^
Oeno1	10.30 ± 0.87 ^a^	12.49 ± 0.41 ^a^	\	0.21 ± 0.01 ^b^	11.41 ± 0.88 ^a^	6.03 ± 0.08 ^a^	\	40.17 ± 2.11 ^a^
Q19	8.76 ± 1.27 ^ab^	10.73 ± 1.01 ^b^	\	\	11.27 ± 3.97 ^a^	3.11 ± 0.21 ^b^	\	33.34 ± 6.50 ^a^

Note: ^a^ and ^b^ indicate significance analysis, and “ND” indicates that the biogenic amine component was not detected. Tested within column.

## Data Availability

Data is contained within the article.

## Appendix A

**Table A1:** Aroma type and content.

Volatile Components (Aromas)	Threshold/(μg/L)	Aroma Characteristics	Aroma Substance Content/(μg/L)		
			Non-MLF	Q19	Oeno1
Alcohol					
1-Butanol, 3-methyl-	\	\	4.7 ± 0.5 ^a^	ND	ND
2,3-Butanediol	120,000	Rubber, cream, fruit	20.6 ± 15.7 ^a^	29.0 ± 15.9 ^a^	46.1 ± 19.4 ^a^
1-Hexanol	8000	Grass, raisins	112.6 ± 11.6 ^a^	120.0 ± 1.6 ^a^	99.6 ± 3.9 ^a^
1-Propanol, 3-(methylthio)-	1000	Raw potato, garlic	6.7 ± 3.8 ^a^	6.0 ± 1.8 ^a^	3.0 ± 1.4 ^a^
1-Octanol	800	Jasmine, lemon	27.2 ± 3 ^a^	38.9 ± 3.8 ^a^	33.1 ± 4.7 ^a^
Phenylethyl Alcohol	1100	Rose, pollen	601.1 ± 161.5 ^a^	736.0 ± 155.3 ^a^	428.0 ± 79.8 ^a^
5-Nonanol, 5-methyl-	\	\	9.5 ± 0 ^a^	10.9 ± 0.60 ^a^	10.1 ± 1.3 ^a^
1-Nonanol	\	\	37.8 ± 7.4 ^a^	45.3 ± 0.9 ^a^	32.2 ± 7.9 ^a^
1-Decanol	500	Orange blossom	33.3 ± 5.7 ^a^	36.2 ± 9.0 ^a^	32.1 ± 8.5 ^a^
1-Dodecanol	1000	Floral	3.5 ± 0.4 ^a^	4.1 ± 0.2 ^a^	3.2 ± 0.4 ^a^
1,6,10-Dodecatrien-3-ol, 3,7,11-trimethyl-, (E)-	\	\	2.4 ± 0.6 ^a^	6.1 ± 1.1 ^a^	2.7 ± 1.0 ^a^
n-Nonadecanol-1	\	\	3.7 ± 0.2 ^a^	5.2 ± 2.6 ^a^	5.9 ± 2.1 ^a^
2,6,10-Dodecatrien-1-ol, 3,7,11-trimethyl-	\	Floral	9.0 ± 1.2 ^b^	7.6 ± 2.1 ^a^	ND
n-Tridecan-1-ol	\	\	4.2 ± 1.8 ^a^	ND	ND
3-Ethyl-4-methylpentan-1-ol	\	\	ND	3.8 ± 0 ^a^	3.5 ± 0 ^a^
1-Hexanol, 2-ethyl-	\	\	ND	14.4 ± 2.5 ^a^	9.4 ± 1.2 ^a^
2,6-Octadien-1-ol, 3,7-dimethyl-, (Z)-	\	Rose, citrus	ND	7.5 ± 0.4 ^a^	5.6 ± 1.5 ^a^
Content/type			876.8/14	1101.0/15	719.3/14
Esters	\	\			
1-Butanol, 3-methyl-, acetate	200	Fruity, fresh banana	181.1 ± 11.2 ^a^	121.4 ± 13.3 ^b^	178.4 ± 7.3 ^a^
Hexanoic acid, ethyl ester	14	Green apple, fruity, strawberry	536.6 ± 67.6 ^a^	540.2 ± 9.5 ^a^	417.7 ± 74.1 ^a^
Octanoic acid, methyl ester	200	Citrus	2.7 ± 0.9 ^a^	3.6 ± 0.2 ^a^	3.3 ± 0.5 ^a^
Butanedioic acid, diethyl ester	1200	Wine curve	37.2 ± 16.8 ^b^	407.1 ± 43.9 ^a^	149.0 ± 27.9 ^b^
Octanoic acid, ethyl ester	250	Fruity, aniseed	1078 ± 304.7 ^a^	1256.1 ± 20.5 ^a^	1079.1 ± 207.6 ^a^
Nonanoic acid, ethyl ester	1300	Rose, fruity	2.4 ± 0.9 ^a^	3.1 ± 0 ^a^	2.4 ± 0.4 ^a^
Decanoic acid, methyl ester	\	Sweet fruit	3.4 ± 0.5 ^a^	4 ± 0.1 ^a^	3.7 ± 0.9 ^a^
Ethyl 9-decenoate	100	\	8.9 ± 2.5 ^a^	10.4 ± 0.7 ^a^	7.8 ± 1.4 ^a^
Decanoic acid, ethyl ester	200	Fatty, fruity	434.6 ± 124.3 ^a^	523.7 ± 10.0 ^a^	397.5 ± 77.2 ^a^
Octanoic acid, 3-methylbutyl ester	125	Floral, fruity	2.7 ± 0.9 ^a^	3.1 ± 0.2 ^a^	2.5 ± 0.5 ^a^
Dodecanoic acid, methyl ester	\	\	1.5 ± 0.6 ^a^	ND	0.5 ± 0.2 ^a^
Ethyl 3-hydroxyoctadecanoate	\	\	1.6 ± 0.5 ^a^	ND	ND
Dodecanoic acid, ethyl ester	1500	Floral, fruity	73.3 ± 24.8 ^a^	52.5 ± 3.3 ^a^	41.1 ± 11.8 ^a^
Pentadecanoic acid, 3-methylbutyl ester	3000	Rose	1.7 ± 0.1 ^a^	1.3 ± 0.1 ^a^	ND
Tetradecanoic acid, ethyl ester	2000	Floral, cinnamon	7.7 ± 1.3 ^a^	3.8 ± 0.4 ^b^	ND
Isopropyl myristate	\	\	3.7 ± 1.4 ^a^	2.2 ± 0.1 ^a^	2.7 ± 1.5 ^a^
Hexadecanoic acid, ethyl ester	1500	\	21.1 ± 5.5 ^a^	ND	ND
2(3H)-Furanone, 5-dodecyldihydro-	\	\	1.0 ± 0.1 ^a^	ND	ND
Octadecanoic acid, ethyl ester	\	\	0.5 ± 0.2 ^a^	0.5 ± 0.1 ^a^	ND
Hexanoic acid, 2-ethyl-, hexadecyl ester	\	\	0.2 ± 0.1 ^a^	ND	ND
Acetic acid, hexyl ester	\	\	ND	2.6 ± 0 ^a^	2.4 ± 0 ^a^
2-Hexenoic acid, ethyl ester	\	\	ND	4.3 ± 0 ^a^	4.0 ± 0.4 ^a^
Pentanoic acid, 2-hydroxy-4-methyl-, ethyl ester	\	\	ND	12.3 ± 0 ^a^	4.8 ± 0.3 ^b^
Isoamyl lactate	\	Fruity, caramel	ND	16.8 ± 0.1 ^a^	7.9 ± 0.5 ^b^
Succinic acid, butyl ethyl ester	\	\	ND	5.8 ± 1.2 ^a^	2.7 ± 1.0 ^a^
Butyrolactone	\	Butter, caramel	ND	0.6 ± 0.1 ^a^	ND
Butanedioic acid, ethyl 3-methylbutyl ester	\	\	ND	75.2 ± 13.7 ^a^	46.7 ± 13.6 ^b^
n-Caprylic acid isobutyl ester	\	\	ND	0.6 ± 0 ^a^	ND
Succinic acid, ethyl octyl ester	\	\	ND	17.3 ± 3.8 ^a^	ND
Hexadecanoic acid, ethyl ester	1500	Fatty	ND	11.6 ± 2.5 ^a^	8.5 ± 2.4 ^b^
Isopropyl palmitate	\	\	ND	6.0 ± 4.6 ^a^	3.8 ± 2.1 ^b^
Dodecanoic acid, isooctyl este	\	\	ND	1.9 ± 0.8 ^a^	ND
Content/type	\	\	2400.4/20	3089.3/27	2366.9/21
Acids					
Octanoic acid	500	Cheese, astringency	148.8 ± 62.4 ^a^	149.8 ± 44.7 ^a^	92.5 ± 15.2 ^a^
n-Hexadecanoic acid	\	\	35.2 ± 34.4 ^a^	97.3 ± 22.6 ^a^	1.9 ± 0.1 ^a^
Tetradecanoic acid	\	\	3.1 ± 0.5 ^b^	25.3 ± 6.2 ^a^	1.5 ± 0.5 ^b^
Octadecanoic acid	\	\	6.4 ± 0.1 ^a^	ND	ND
n-Decanoic acid	1000	Fatty	86.3 ± 49.9 ^a^	117.5 ± 58.8 ^a^	89.0 ± 20.0 ^a^
Dodecanoic acid	1500	Laurel oil	ND	26.6 ± 1.7 ^a^	16.5 ± 4.6 ^a^
3-(2,2,4-Trimethylcyclohex-3-enyl)prop-2-enoic acid	\	\	ND	187.6 ± 15.6 ^a^	186.3 ± 1.5 ^a^
6-Octadecenoic acid, (Z)-	\	\	ND	3.7 ± 3.0 ^a^	3.2 ± 0.2 ^a^
Oleic Acid	\	\	ND	3.7 ± 1.0 ^b^	12.6 ± 10.0 ^a^
Pentadecanoic acid	\	\	ND	1.1 ± 0.7 ^b^	43.7 ± 33.7 ^a^
Heptadecanoic acid	\	\	ND	2.1 ± 0.4 ^a^	1.4 ± 0.1 ^a^
Tridecanoic acid	\	\	ND	2.6 ± 0.5 ^a^	ND
Octadecanoic acid	\	\	ND	21.5 ± 13.5 ^a^	ND
2-Ethyl-2-hydroxybutyric acid	\	\	ND	0.0033 ± 0.0002 ^a^	ND
4-Vinylbenzoic acid	\	\	ND	1.7 ± 0.9 ^a^	ND
Content/type			280/5	644.7/14	448.9/10
Terpenoids					
5,9-Undecadien-2-one, 6,10-dimethyl-	\	\	8.1 ± 4.8 ^b^	10.4 ± 4.8 ^a^	8.0 ± 4.3 ^b^
2-Buten-1-one, 1-(2,6,6-trimethyl-1,3-cyclohexadien-1-yl)-	\	\	ND	20.8 ± 20.4 ^a^	ND
2-Buten-1-one, 1-(2,6,6-trimethyl-1,3-cyclohexadien-1-yl)-, (E)-	\	Honey, rose	ND	15.4 ± 14.9 ^a^	ND
Citronellol	200	Rose, lime	16 ± 4.1 ^a^	18.1 ± 2.8 ^a^	12 ± 3.9 ^a^
Linalool	\	\	ND	ND	4.5 ± 0.9 ^a^
Squalene	\	\	ND	3.7 ± 2.9 ^a^	1.9 ± 1.3 ^b^
Content/type	\	\	24.1/2	68.4/5	26.4/4
Aldehydes and ketones	\	\			
Tetradecanal	\	\	0.7 ± 0.2 ^a^	ND	ND
Decanal	10	Bitterness	28.0 ± 15.8 ^a^	20.4 ± 6.7 ^a^	17.1 ± 8.8 ^a^
4-Octanone	\	\	64.9 ± 7.9 ^a^	63.9 ± 4.5 ^a^	65.4 ± 5.4 ^a^
Pentadecanal-	\	\	ND	0.7 ± 0.1 ^a^	ND
Content/type	\	\	93.6/3	85.0/3	82.5/2
Others					
2,6,10-Trimethyltridecane	\	\	6.0 ± 0.3 ^a^	5.5 ± 0.5 ^a^	3.0 ± 2.1 ^b^
Octane, 1,1′-oxybis-	\	\	ND	5.3 ± 1.1 ^b^	10.8 ± 1.2 ^a^
Undecane, 3,6-dimethyl-	\	\	ND	ND	6.7 ± 0 ^a^
Heptylcyclohexane	\	\	2.9 ± 0.2 ^a^	ND	ND
2-Methylhexacosane	\	\	1.2 ± 0.3 ^a^	ND	ND
3-(2,2,4-Trimethylcyclohex-3-enyl)prop-2-enoic acid	\	\	203.4 ± 12.3 ^a^	ND	ND
4-Heptanol, 2,6-dimethyl-4-propyl-	\	\	9.1 ± 0.2 ^a^	ND	ND
Content/type			222.6/5	10.8/2	23.2/4

Note: ^a^ and ^b^ indicate significance analysis. Contents are recorded as 4-octanol. Different lowercase letters in the same row indicate significant differences (*p* < 0.05). “ND” indicates that the volatile component was not detected, and “\” indicates that the relevant literature was not consulted.

**Table A2:** Aroma type and content.

Volatile Components (Aromas)	Threshold /(μg/L)	Aroma Characteristics	Aroma Substance Content/(μg/L)		
			Non-MLF	Q19	Oeno1
Alcohols					
1-Butanol, 3-methyl-	7000	Bitter almond, alcohol	3.8 ± 0.4 ^a^	5.9 ± 0 ^a^	7.2 ± 1.3 ^a^
1-Hexanol	\	\	125.5 ± 7.3 ^b^	150.3 ± 9.6 ^ab^	159.7 ± 0.1 ^a^
3-Ethyl-4-methylpentan-1-ol	\	\	4.3 ± 0.2 ^b^	7.7 ± 0.7 ^a^	9.0 ± 0.3 ^a^
1-Hexanol, 2-ethyl-	\	\	7.7 ± 0.8 ^a^	9.3 ± 2.9 ^a^	13.8 ± 0.8 ^a^
1-Octanol	800	Jasmine, lemon	27.4 ± 2.3 ^a^	103.6 ± 73.8 ^a^	32.7 ± 0.2 ^a^
Phenylethyl Alcohol	1100	Rose, pollen	740.3 ± 121.4 ^a^	337.1 ± 327.0 ^a^	546.8 ± 172.4 ^a^
1-Nonanol	\	\	40.0 ± 3.7 ^a^	45.5 ± 2.5 ^a^	51.1 ± 2.2 ^a^
1-Dodecanol	1000	Floral	3.2 ± 0.7 ^a^	3.1 ± 0.2 ^a^	3.2 ± 0.5 ^a^
1,6,10-Dodecatrien-3-ol, 3,7,11-trimethyl-, (E)-	\	\	0.9 ± 0 ^c^	3.9 ± 0.6 ^b^	6.3 ± 0.3 ^a^
n-Nonadecanol-1	\	\	2.0 ± 0.2 ^a^	30.5 ± 27.2 ^a^	2.5 ± 0.5 ^a^
n-Tridecan-1-ol	\	\	1.2 ± 0.3 ^a^	ND	ND
2,3-Butanediol	\	Rubber, cream, fruit	ND	15.5 ± 1.8 ^b^	22.1 ± 9.3 ^a^
2,6,10-Dodecatrien-1-ol, 3,7,11-trimethyl-	\	\	ND	ND	7.9 ± 0.4 ^a^
4-Heptanol, 2,6-dimethyl-4-propyl-	\	\	ND	ND	6.2 ± 2.1 ^a^
5-Nonanol, 5-methyl-	\	\	ND	ND	10.0 ± 0.2 ^a^
Content/type			956.6/11	712.9/12	878.9/14
Esters					
1-Butanol, 3-methyl-, acetate	200	Fruity, fresh banana	91.0 ± 1.1 ^a^	179.3 ± 20.5 ^a^	127.1 ± 28.9 ^a^
Hexanoic acid, ethyl ester	14	Green apple, fruity, strawberry	537.0 ± 30.4 ^a^	554.1 ± 10.2 ^a^	554.2 ± 25.5 ^a^
Acetic acid, hexyl ester	2	Pear fruit	2.4 ± 0.1 ^a^	3.0 ± 0.4 ^a^	3.0 ± 0.2 ^a^
2-Hexenoic acid, ethyl ester	80	Banana, green apple	5.5 ± 0.3 ^a^	6.6 ± 0.3 ^a^	5.7 ± 1.0 ^a^
Octanoic acid, methyl ester	200	Citrus	3.6 ± 0.4 ^a^	2.9 ± 0.6 ^a^	4.8 ± 0 ^a^
Butanedioic acid, diethyl ester	1200	Rich, wine	38.9 ± 3.8 ^c^	176.1 ± 4.9 ^b^	256.4 ± 20.4 ^a^
Octanoic acid, ethyl ester	250	Fruity, aniseed	1014.2 ± 104.6 ^a^	1085.9 ± 27 ^a^	1370.2 ± 36.7 ^a^
Nonanoic acid, ethyl ester	1300	Rose, fruity	2.6 ± 0.5 ^b^	4.0 ± 0.1 ^ab^	5.3 ± 0.1 ^a^
Decanoic acid, methyl ester	\	Sweet fruit	ND	3.9 ± 0.1 ^b^	4.8 ± 0.6 ^a^
Succinic acid, butyl ethyl ester	\	\	ND	3.3 ± 0.3 ^a^	ND
Succinic acid, ethyl octyl ester	\	\	ND	ND	6.1 ± 0.9 ^a^
Decanoic acid, ethyl ester	200	Fatty, fruity	349.2 ± 76.9 ^b^	536.3 ± 2.9 ^ab^	732.7 ± 13.4 ^a^
Butanedioic acid, ethyl 3-methylbutyl ester	\	\	2.9 ± 0.1 ^c^	63.9 ± 1.4 ^b^	98.0 ± 10.2 ^a^
Octanoic acid, 3-methylbutyl ester	125	Floral, fruity	1.5 ± 0.6 ^a^	2.7 ± 0 ^a^	6.9 ± 2.0 ^a^
Dodecanoic acid, ethyl ester	1500	Floral, fruity	24.4 ± 6.1 ^b^	34.4 ± 7.5 ^ab^	51.1 ± 0.1 ^a^
Isopropyl myristate		Floral, cinnamon	4.0 ± 2.4 ^a^	1.5 ± 0.4 ^a^	2.2 ± 0.4 ^a^
Hexadecanoic acid, ethyl ester	1500	Fatty	11.4 ± 1.3 ^ab^	6.9 ± 0.6 ^b^	20.9 ± 3.4 ^a^
Isopropyl palmitate	\	\	7.2 ± 0.5 ^a^	3.7 ± 0.7 ^b^	ND
Dodecanoic acid, isooctyl ester	\	Floral, fruity	1.1 ± 0.1 ^a^	ND	1.2 ± 0.1 ^a^
Hexanoic acid, 2-ethyl-, hexadecyl ester	\	\	0.1 ± 0 ^a^	0.4 ± 0.2 ^a^	0.4 ± 0.2 ^a^
Acetic acid, 2-phenylethyl ester	250	Rose, jasmine	30.5 ± 4.5 ^a^	ND	ND
Pentanoic acid, 2-hydroxy-4-methyl-, ethyl ester	\	\	ND	8.0 ± 4.4 ^b^	11.6 ± 2.3 ^a^
2-Propenoic acid, 3-(4-methoxyphenyl)-, 2-ethylhexyl ester	\	\	ND	26.1 ± 24.0 ^a^	ND
Ethyl 9-hexadecenoate	\	\	ND	ND	2.4 ± 0.3 ^a^
Octadecanoic acid, ethyl ester	\	\	ND	0.5 ± 0.1 ^a^	ND
Content/type			2128.0/18	2703.9/21	3265.5/20
Acids					
Octanoic acid	500	Cheese, astringency	131.5 ± 21.9 ^a^	131.4 ± 2.7 ^a^	214.1 ± 24.6 ^a^
3-(2,2,4-Trimethylcyclohex-3-enyl)prop-2-enoic acid	\	\	198.5 ± 6.0 ^a^	280.5 ± 79.1 ^a^	204.5 ± 2.2 ^a^
n-Decanoic acid	1000	Fatty	109.5 ± 39.7 ^a^	142.0 ± 1.4 ^a^	240.5 ± 30.8 ^a^
Dodecanoic acid	1500	Laurel oil	16.1 ± 2.3 ^a^	20.4 ± 1.0 ^a^	20.8 ± 4.5 ^a^
n-Hexadecanoic acid	\	\	15.6 ± 14.0 ^a^	1.8 ± 0.5 ^a^	24.9 ± 23.9 ^a^
Myristic acid	\	\	1.6 ± 0.4 ^a^	ND	ND
Tetradecanoic acid	\	\	ND	7.4 ± 4.9 ^a^	ND
Oleic Acid	\	\	ND	15.5 ± 11.8 ^a^	ND
Pentadecanoic acid	\	\	ND	76.2 ± 25.3 ^a^	ND
Content/type			473.1/6	675.6/8	705.1/5
Terpenoids					
5,9-Undecadien-2-one, 6,10-dimethyl-	\	\	16.4 ± 5.3 ^a^	3.6 ± 0.3 ^a^	3.4 ± 0.1 ^a^
Citronellol	200	Rose, lemon	13.3 ± 3.3 ^a^	12.0 ± 0 ^a^	15.2 ± 0.3 ^a^
Squalene	\	\	1.0 ± 0.4 ^a^	32.2 ± 21.1 ^a^	0.8 ± 0 ^a^
Content/type			30.7/3	47.8/3	19.4/3
Aldehydes and ketones					
4-Octanone	\	\	63.8 ± 0.3 ^a^	117.0 ± 51.0 ^a^	58.5 ± 0.8 ^a^
Decanal	\	\	13.2 ± 1.4 ^a^	11.9 ± 2.4 ^a^	7.9 ± 0.7 ^a^
2-Pentadecanone, 6,10,14-trimethyl-	\	\	0.6 ± 0.1 ^a^	0.4 ± 0.1 ^a^	0.9 ± 0.1 ^a^
Content/type			77.7/3	128.6/3	76.5/3
Others					
Heptylcyclohexane	\	\	2.5 ± 0.4 ^a^	ND	ND
2,6,10-Trimethyltridecane	\	\	4.5 ± 0.4 ^a^	5.3 ± 0.6 ^a^	5.7 ± 0.8 ^a^
Octane, 1,1′-oxybis-	\	\	8.1 ± 4.0 ^a^	29.7 ± 26.8 ^a^	13.0 ± 10.0 ^a^
2-Methylhexacosane	\	\	1.2 ± 0.5 ^a^	ND	ND
Content/type			16.5/4	35.2/2	18.8/2

Note: ^a^, ^b^ and ^c^ indicate significance analysis. Contents are recorded as 4-octanol. Different lowercase letters in the same row indicate significant differences (*p* < 0.05). “ND” indicates that the volatile component was not detected, and “\” indicates that the relevant literature was not consulted.
